# Association between Body Composition Contents and Hepatic Fibrosis in Sarcopenic Obesity

**DOI:** 10.3390/jcm12134279

**Published:** 2023-06-26

**Authors:** Tae-Hoon Kim, Chang-Won Jeong, ChungSub Lee, SiHyeong Noh, Dong Wook Lim, Jin Woong Kim, Hyung Joong Kim, Youe Ree Kim

**Affiliations:** 1 Medical Convergence Research Center, Wonkwang University, Wonkwang University Hospital, Iksan 54538, Republic of Korea; cslee99@gmail.com (C.L.); nosij123@wku.ac.kr (S.N.); dwl316@wku.ac.kr (D.W.L.); 2 Department of Radiology, Chosun University Hospital of Medicine, Chosun University College, Gwangju 61453, Republic of Korea; radjin@chosun.ac.kr; 3 Department of Biomedical Engineering, Kyung Hee University, Dongdaemun-gu, Seoul 02447, Republic of Korea; bmekim@khu.ac.kr; 4 Department of Radiology, Wonkwang University Hospital, Wonkwang University School of Medicine, Iksan 54538, Republic of Korea; sweetynn@naver.com

**Keywords:** sarcopenic obesity (SO), hepatic fibrosis, body composition, third lumber spine (L3)

## Abstract

It is well established that sarcopenic obesity (SO) is linked to many diseases such as metabolic and non-alcoholic fatty liver diseases, but there is little known about the relationship between SO and hepatic fibrosis progression in chronic liver disease. This study compared body composition contents in patients with non-obesity (NOb) and SO using abdominal magnetic resonance imaging and investigated the relationship between hepatic fibrosis and SO factors. This retrospective study enrolled 60 patients (28 NOb; 32 SO) from June 2014 to December 2020. Patients underwent histopathologic investigation where they classified fibrosis stages based on the Meta-analysis of Histological Data in Viral Hepatitis fibrosis scoring system. Muscle and fat areas at the third lumber vertebra level were assessed. The variation in the areas of muscle (MA), subcutaneous adipose tissue (SAT), and visceral adipose tissue (VAT) among fibrosis stages, and associations between hepatic fibrosis and SO factors, were analyzed. There were significant differences in SAT and VAT (*p* < 0.001), whereas there was no difference in MA (*p* = 0.064). There were significant differences in MA/SAT (*p* = 0.009), MA/VAT (*p* < 0.001), and MA/(SAT+VAT) (*p* < 0.001). In all the patients, hepatic fibrosis positively correlated with serum aspartate aminotransferase level (AST, *R* = 0.324; *p* = 0.025). Especially in SO patients, hepatic fibrosis closely correlated with body mass index (BMI, *R* = 0.443; *p* = 0.011), AST (*R* = 0.415; *p* = 0.044), VAT (*R* = 0.653; *p* < 0.001), MA/VAT (*R* = −0.605; *p* < 0.001), and MA/(SAT+VAT) (*R* = −0.416; *p* = 0.018). However, there was no association in NOb patients. This study demonstrated that SO patients had larger SAT and VAT than NOb patients. Hepatic fibrosis in SO positively correlated with body visceral fat composition in combination with BMI and AST level. These findings will be useful for understanding the relationship between the hepatic manifestation of fibrosis and body fat composition in sarcopenia and SO.

## 1. Introduction

The terminology “sarcopenic obesity (SO)” has been proposed to identify obesity with low muscle strength, skeletal muscle mass (SMM), and physical performance [1]. Sarcopenic obesity is a multifactorial condition characterized by the co-occurrence of sarcopenia and obesity and has a synergistically adverse effect in aggravating metabolic and cardiovascular diseases and mortality [2,3]. The definitions and diagnostic criteria for SO combine sarcopenia (the International Classification of Diseases 10th Revision (ICD-10-CM) with the European Society for Clinical Nutrition and Metabolism (ESPEN) and the European Association for the Study of Obesity (EASO) consensus statements) [4,5] as defined through variable criteria with obesity, determined by either body mass index (BMI) or excess adiposity levels [6,7,8,9]. This vicious cycle may continue because the fat accumulation within the organs and tissues of the human body and the decrement in muscle mass are interdependent. Recently, there has been a growing interest in SO and/or sarcopenia, noted as a crucial health risk [10,11,12].

In the assessment of sarcopenia and SO, the importance of evaluating body composition contents as muscle mass (MM) and fat mass (FM) is indisputable. Among the composition contents, measuring SMM is important because MM is involved in energy homeostasis and fatty acid oxidation and is a critical determinant for insulin-mediated glucose metabolism in the whole body [13]. Current image-based quantitative assessments of SO and/or sarcopenia are performed using dual-energy X-ray absorptiometry (DXA) [14], computed tomography (CT) [15], and magnetic resonance imaging (MRI) [16]. For CT and MRI scans, unlike DXA measuring bone mineral density, the analysis of image slices at the third lumbar vertebra (L3) is the best compromise site for assessing the total volume of skeletal muscle, visceral adipose tissue, and subcutaneous adipose tissue in both sexes [17]. Compared with CT, the MR imaging modality provides superior soft tissue contrast in the liver, kidney, muscle, adipose tissue, nerve, ligaments, and so on, and does not cause ionizing radiation effects in patients [18,19,20]. However, the study of MRI remains relatively insufficient as measurement methods and diagnostic criteria for SMM have not yet been established. To quantify body composition contents from a single MRI scan, a research group recently developed open-source ImageJ-based sarcopenia quantification software, providing a rapid processing time through simplified quantitative analysis for easy clinical implementation [16]. Fat and muscle area indices quantified using developed sarcopenic software are applicable for comparing the difference between SO and healthy control groups, but it is unclear whether the specific mechanism underlying the sarcopenic pathological responses involved in the decrease in MM is obesity and aging [21,22]. Thus, through the quantitative assessment of body composition contents, the investigations focusing on the prevalence and health risks of sarcopenia in patients with obesity are important topics for preventive purposes.

Several studies have reported that SO is observationally associated with elevated BMI [23,24], functional impairment, physical disorders (skeletal weakness and fragility), metabolic complications, a reduction in quality of life [25], disease incidence [26,27], and increased mortality [28,29]. The main factors that counteract the muscle loss are diet [30,31] and exercise [32,33,34]; these factors are considered in that a lower MM is related to a higher risk of non-alcoholic fatty liver disease (NAFLD) [35,36]. These studies indicate that more patients within the population with obesity have an increased FM or weakened musculoskeletal system in all age groups, and the prevalence and risks of SO have increased in the presence of diseases such as fatty liver, hepatic fibrosis, metabolic syndrome (possibly has a causative role in hepatic pathogenesis), and cardiovascular disease [23,24,27]. Furthermore, recent studies have investigated the impacts of fat accumulation and progression within the liver and their associations with sarcopenia [13,37,38,39,40,41]. Skeletal muscle plays a crucial role in fatty liver oxidation, the transport and disposal of glucose, and energy homeostasis, which are all key determinants in the pathophysiology of liver diseases [37]. These mechanisms drive ectopic fat accumulation in the liver (fatty liver, steatosis) and muscle (myosteatosis) [13]. Myosteatosis (rather than MM) seems to be closely associated with the severity of liver injury [38]. Additionally, fibrosis in NAFLD is associated with more indices of FM than those of MM [39]. In the factors promoting liver damage and fibrosis, the role of mitochondria and its dysfunction has been linked to the progression of NAFLD. Thus, many strategies to prevent or restore liver function focus on the improvement in mitochondrial activities [42,43,44]. Consequently, the FM should be controlled to prevent NAFLD progression [39]. All of these studies suggested that fatty liver and sarcopenia commonly share physiological pathways and are interconnected via the muscle–liver–adipose tissue axis. The axis for muscle–liver–adipose tissue has an important role in the changes in the body’s composition, resulting in a discrete phenotype that enables the identification of the fatty liver disease phenotype [40]. Thus, it indicates that SO may trigger worse clinical outcomes, including non-alcoholic fatty liver and hepatic fibrosis progression in conjunction with musculoskeletal disabilities. However, mechanisms underlying the expansion of sarcopenic pathological responses involved in the fibrotic progression of chronic hepatic diseases in patients with SO are still unclear.

Here, with the help of simplified quantitative analysis, we investigated the association between SO and hepatic fibrosis in chronic liver disease using ImageJ-based sarcopenia quantification software. This study compared body composition contents in patients with non-obesity and SO using abdominal MRI and investigated the relationship between hepatic fibrosis and SO factors.

## 2. Subjects and Methods

### 2.1. Ethics Statement

The local institutional review board (IRB) approved this retrospective study (Wonkwang University Hospital (WKUH), No. 2018-01-005), and the requirements for written informed consent were exempted. WKUH IRB committee approved the use of anonymous archival data (MRI and electronic health records). This study was performed in compliance with the Declaration of Helsinki and Good Clinical Practice.

### 2.2. Patient Population

Consecutive patients from June 2014 to December 2020, who were over 20 years of age, who underwent abdominal MRI, and who had available histopathologic information and serologic test results were retrospectively identified. All patients were included with serum chemistry data for metabolic and inflammatory status. For the classification of liver fibrosis and obesity, liver fibrosis was confirmed from pathologic information, and the Korean standard body mass index (BMI, kg/m^2^) was used as the selection criterion, with a BMI cut-off value of 25.0 kg/m^2^ [45]. Together with the liver fibrosis stage, a total of 60 patients, including 28 with NOb (mean age: 58.0 ± 14.5 years) and 32 with SO (mean age: 54.0 ± 14.9 years), were enrolled (Table 1). They complained of fatigue and inactivity, and they appeared weaker in terms of maximum muscular strength [46].

### 2.3. Reference Standard for Diagnosing Liver Fibrosis

**Figure 1:**
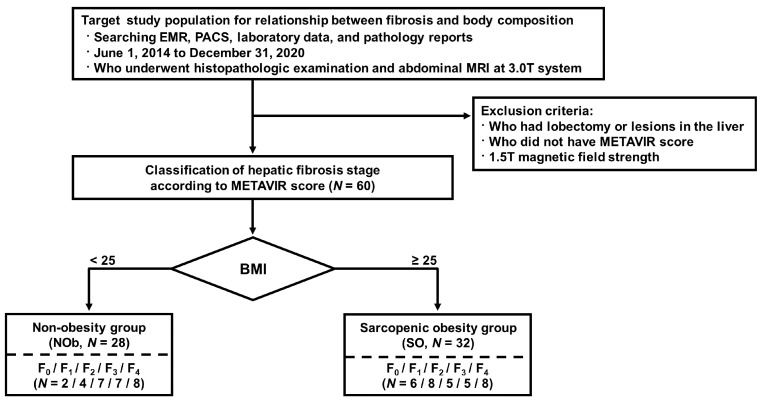
Flowcharts of the study population for inclusion. BMI: body mass index; EMR: electronic medical records; METAVIR: meta-analysis virus hepatitis histological scoring system; PACS: picture archiving and communication system.

All histological information was obtained based on percutaneous needle biopsy or surgical biopsy. A pathologist with 10 years of experience analyzed the histopathological data based on the Meta-analysis of Histological Data in Viral Hepatitis (METAVIR) fibrosis scoring system [47,48]: F0, no fibrosis; F1, portal fibrosis; F2, periportal fibrosis; F3, septal fibrosis; and F4, cirrhosis. The final cohort was divided into five groups (F0–F4) according to liver fibrosis stages as follows: F0 (n = 8), F1 (n = 12), F2 (n = 12), F3 (n = 12), and F4 (cirrhosis, n = 16). The numbers of NOb and SO groups within the final cohort based on the F0–F4 classification were as follows: F0 (Total/NOb/SO; n = 8/2/6), F1 (n = 12/4/8), F2 (n = 12/7/5), F3 (n = 12/7/5), and F4 (cirrhosis, n = 16/8/8) (Figure 1).

### 2.4. Magnetic Resonance Imaging for Abdomen

Abdominal MRI scans were acquired using a 3 Tesla Achieva MRI system (Philips Healthcare, Best, The Netherlands) with an array coil with 32 receiver channels. The THRIVE images were obtained with the following parameters: repetition time (TR)/echo time (TE) = 4.5/1.98 ms, the field of view = 38 × 38 × 14 cm^3^, the number of excitations = 2, slice thickness = 0.74 × 0.74 × 2.0 mm^3^, the number of slices = 100, matrix size = 512 × 512 pixels, and scan time = 16 s.

### 2.5. Measurement of Body Compositions on Third Lumbar Vertebra MRI

**Figure 2:**
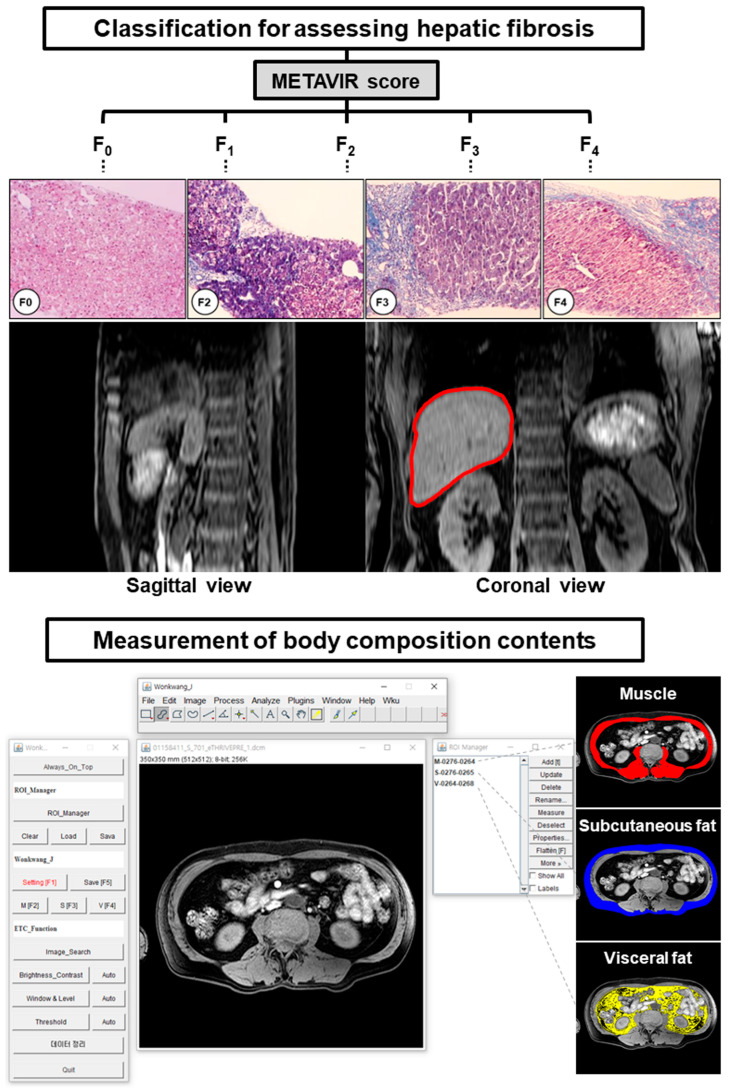
Hepatic fibrosis classification based on the Meta-analysis of Histological Data in Viral Hepatitis (METAVIR) fibrosis scoring system (liver inside red line on the upper panel) and representative measurement of body composition contents (muscle, subcutaneous adipose tissue, and visceral adipose tissue) from abdominal MR scans at third lumbar vertebra level (L3, lower panel). The graphic user interface (GUI) of the developed software “sarcopenia plug-in” including ImageJ platform basic menu bar, sarcopenia plug-in window (left in the lower panel), and region of interest (ROI) manager window (right in the lower panel). An example demonstrating the ROI extraction for quantifying muscle area (red area), subcutaneous adipose tissue (blue area), and visceral adipose tissue (yellow area) mass in a patient with sarcopenic obesity using “sarcopenia plug-in”. The original ImageJ software (ver.1.51t, Java 1.8.0_191 64 bits; the National Institutes of Health (NIH), Bethesda, MD, USA) is available at https://imagej.nih.gov/ij/, accessed on 31 December 2017.

To measure body composition contents as muscle and fat mass, this study used a single-slice abdominal MRI analysis with a cross-sectional study design [17,49]. The third lumbar vertebra (L3) level image was chosen as the position for quantitative analysis because the level identified various anatomical areas (the intestines, kidneys, liver, and spine) and the seven major muscles (the erector spinae, external and internal obliques, psoas, quadratus lumborum, transversus abdominus, and rectus abdominus) (Figure 2) [17,50]. Therefore, it is the most proper position to analyze the association between various conditions and diseases, including of aging, obesity, and sarcopenia [51].

### 2.6. Data Processing and Quantification of Magnetic Resonance Imaging Scans

In order to quantify the body composition contents in the NOb and SO groups, the quantification sarcopenia-specialized software on the ImageJ multiplatform program (ver.1.51t, Java 1.8.0_191 64 bits, the National Institutes of Health (NIH), Bethesda, MD, USA) was used [52]. The processing procedures for MRI data were comprised of following four steps: execution, setting, confirmation, and extraction.

The detailed processes were described in a prior report [16]. Briefly, the MRI data were loaded onto the sarcopenia program, and an L3 level image was chosen from the axial abdominal MRI in each patient to identify the regions of interest (ROIs) of muscle area (MA), subcutaneous adipose tissue (SAT), and visceral adipose tissue (VAT) in the execution step. In the setting step, the selected MRI scan was used to set the window leveling and threshold values, and their values were applied to the opened MRI data. After the setting, the ROIs (MA, SAT, VAT) were manually drawn on the MRI scan using the drawing tools. In the confirmation step, the ROIs of the body composition contents were confirmed by two physicians (J.W.K, Y.R.K), and then, the final ROIs were generated from the overlaid areas between the confirmed ROIs and the regions within the threshold value. In the extraction step, the quantification results from final ROIs were extracted as the color-labeled ROI images and CSV files for the quantified volume data (Figure 2).

To compare body composition contents in both NOb and SO groups, the chosen L3 level MRI scans of patients were independently analyzed for the major composition contents (MA, SAT, VAT). They were blinded to the clinical outcome. The overall volume measurements in each patient were calculated as an arithmetic average and standard deviation for the areas.

### 2.7. Statistical Analysis

The abdominal body composition contents based on liver fibrosis stages were compared with the independent two-sample *t*-test using the statistical package for the social sciences program (SPSS ver.20, Chicago, IL, USA). The variation in body composition contents (muscle and fat) in each fibrosis stage was evaluated with the Mann–Whitney U test. The association between pathologic METAVIR fibrosis stages and other factors was assessed using linear polynomial correlation (*R*). Two-sided *p*-values less than 0.05 were considered to indicate statistical significance in all tests.

## 3. Results

### 3.1. Patient Characteristics

The clinical characteristics and average enzyme levels in the SO and non-obesity (NOb) groups are shown in Table 1. The two age-matched groups significantly differed in their BMI values (*p* < 0.001). However, the serum biochemistry involving albumin (*p* = 0.705), alanine aminotransferase (ALT, *p* = 0.713), and aspartate aminotransferase (AST, *p* = 0.293) levels, alkaline phosphatase (ALP, *p* = 0.572), C-reactive protein (CRP, *p* = 0.198), fasting glucose (*p* = 0.255), and gamma-glutamyl transferase (GGT, *p* = 0.307) showed no significant differences between both groups. Thus, the differences in both groups are indicative of the changes in the body composition contents in conjunction with obesity and liver fibrosis.

### 3.2. Patient Classification and Measurements of Body Composition Contents

Based on the METAVIR score for the liver fibrosis stage, the scores of both the NOb and SO groups were classified for statistical analysis as follows: F0 and F1 (NOb/SO; n = 6/14), F2 (n = 7/5), F3 (n = 7/5), and F4 (cirrhosis, n = 8/8). Figure 2 illustrates the METAVIR fibrosis classification and the quantitative analysis of sarcopenia software with an example image for major composition contents. The classified MRI scans were selected at the L3 location and analyzed with sarcopenia software. The major composition contents in all the patients (muscle, visceral adipose tissue, and subcutaneous adipose tissue) were quantified, as shown in Table 2.

### 3.3. Body Composition Contents in Sarcopenic Obesity with Liver Fibrosis

**Figure 3:**
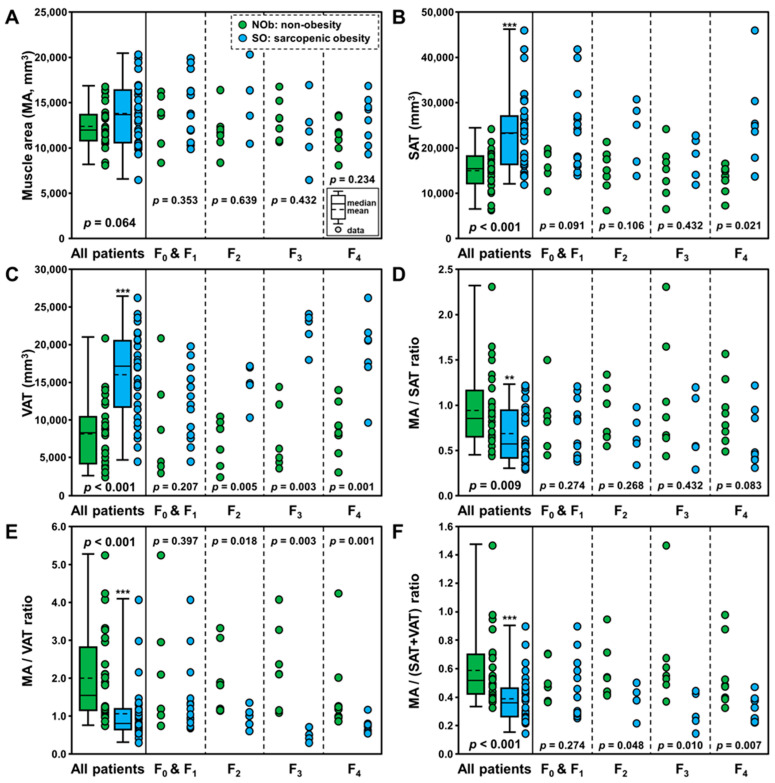
Box plots of the areas of muscle (MA, (**A**)), subcutaneous adipose tissue (SAT, (**B**)), and visceral adipose tissue (VAT, (**C**)), and the ratios of MA/SAT (**D**), MA/VAT (**E**), and MA/(SAT+VAT) (**F**) in non-obesity (NOb) and sarcopenic obesity (SO) groups. Note that asterisks indicate significant differences between NOb and SO according to fibrosis stage as follows: ** *p* < 0.01, and *** *p* < 0.001.

MRI data from 28 patients with SO and 32 patients with NOb were analyzed for the major composition contents using the sarcopenia software. The average areas and ratios of muscle, subcutaneous adipose tissue, and visceral adipose tissue in the two groups are summarized in Table 2. The ratios derived from muscle and fat areas are provided as they are more powerful indexes for discrimination between SO and healthy controls in a previous study. Figure 3 depicts the box plots for the areas of the muscle (MA, Figure 3A), subcutaneous adipose tissue (SAT, Figure 3B), and visceral adipose tissue (VAT, Figure 3C), and the ratios of MA/SAT (Figure 3D), MA/VAT (Figure 3E), and MA/(SAT+VAT) (Figure 3F) in the NOb and SO groups. There were significant differences in SAT (*p* < 0.001) and VAT (*p* < 0.001), whereas there was no difference in MA (*p* = 0.064) between the two groups. Regarding the ratios, two groups were significantly different in MA/SAT (*p* = 0.009), MA/VAT (*p* < 0.001), and MA/(SAT+VAT) (*p* < 0.001). Thus, these ratios are expected to be more powerful indices for discriminating the SO group from the NOb group.

### 3.4. Correlation between Liver Fibrosis and Body Composition Contents in SO

**Figure 4:**
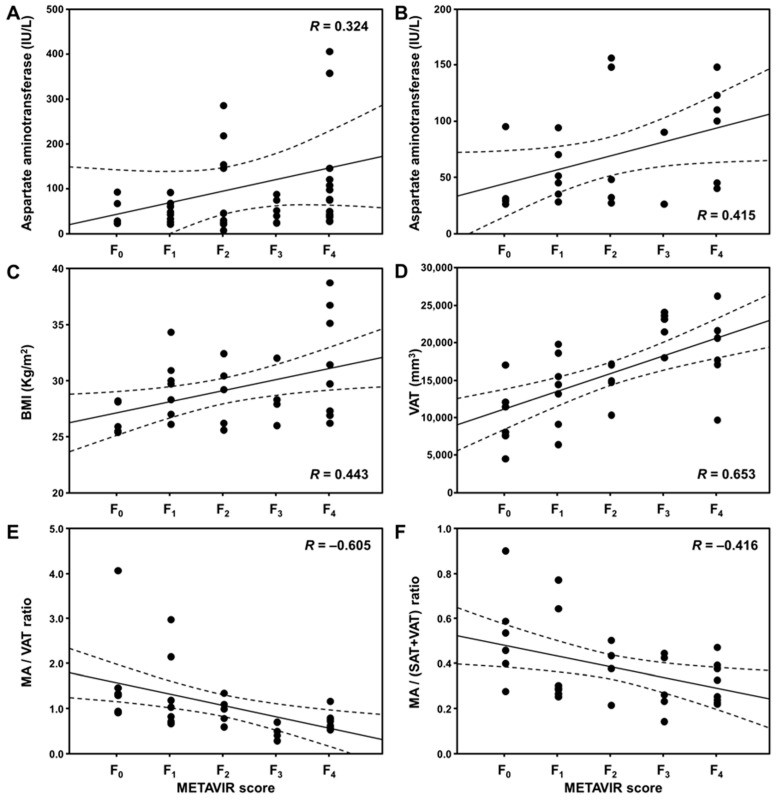
Correlation plots between the pathologic METAVIR scores and other factors. Graphs showing close correlations between AST level and fibrosis stage (F) in all patients (linear polynomial correlation *R* = 0.324; *p* = 0.025) (**A**), and between AST level and F (*R* = 0.415; *p* = 0.044) (**B**), between BMI and F (*R* = 0.443; *p* = 0.011) (**C**), between visceral adipose tissue (VAT) and F (*R* = 0.653; *p* < 0.001) (**D**), between MA/VAT ratio and F (*R* = −0.605; *p* < 0.001) (**E**), and MA/(SAT+VAT) ratio and F (*R* = −0.416; *p* = 0.018) (**F**) within only SO group, respectively. The dashed line in each plot indicates the 95% confidence interval line.

Table 3 lists the correlations between the pathologic METAVIR fibrosis scores and other factors. Figure 4 shows close correlations between the liver fibrosis stage and other factors. In all the patients, hepatic fibrosis positively correlated with serum AST level (linear polynomial correlation *R* = 0.324; *p* = 0.025). Especially in patients with SO, hepatic fibrosis correlated with BMI (*R* = 0.443; *p* = 0.011), AST level (*R* = 0.415; *p* = 0.044), VAT (*R* = 0.653; *p* < 0.001), MA/VAT ratio (*R* = −0.605; *p* < 0.001), and MA/(SAT+VAT) ratio (*R* = −0.416; *p* = 0.018), respectively. The correlation coefficient was the highest between VAT and liver fibrosis stage in patients with SO. However, there was no association in patients with NOb.

## 4. Discussion

This study compared body composition contents in patients with non-obesity and sarcopenic obesity using ImageJ platform-based sarcopenia software. Our study analyzed the difference between patients with SO and NOb by measuring body composition areas from retrospective MRI datasets, which were found to be positively correlated with the liver fibrosis stages and other factors. In this study, abdominal MRI scans with 3-dimensional T1 high-resolution isotropic volume excitation (THRIVE) pulse sequence demonstrated excellent discrimination between patients with SO and those with NOb (as SAT and VAT; *p* < 0.01). Moreover, the ratios of muscle and fat areas that showed better discrimination were MA/SAT (*p* < 0.01), MA/VAT (*p* < 0.001), and MA/(SAT+VAT) (*p* < 0.001), compared to MA, SAT and VAT areas. Thus, these findings demonstrate that the body composition areas and ratios (as muscle, subcutaneous adipose tissue, and visceral adipose tissue) quantified at the L3 level can be useful for diagnosing SO or sarcopenia.

Compared to the original ImageJ program, the present sarcopenia program providing a rapid processing time would be beneficial to clinical implementation, especially for sarcopenia, obesity, and SO. The benefit of the ImageJ software on the basis of Java language is that it is an open-source platform. The program provides high scalability using macro functions and Java plug-in for assessing SO and NOb, as evidenced by the comparison of patients with sarcopenic obesity and healthy controls in a previous study [12]. Future studies could validate the performance efficacy of the sarcopenia program to measure the body composition volumes (muscle, fat) in a variety of liver diseases by using a similar fashion.

With regard to the study design, this retrospective study used criteria for inclusion based on pathologic information, BMI [45], and serum biochemistry for the enrollment of SO and NOb patients. Although our data are histologically proven for the liver fibrosis stage, the retrospective enrollment might be considered for variable factors and/or potential bias. The potential sarcopenic risk factors were as follows: the patient’s selection (age, sex, severity, etc.), clinical conditions (type and dosage of drug for treatment, and so on), and imaging setting (imaging parameters, imaging pulse sequences, type of scanners), and any their combinations might represent risks/bias. In this study, the evaluation method for obesity was the Korean standard BMI (>25.0 kg/m^2^) in association with similar levels of serum enzymes. Image-based body composition quantification in this study is well reflected in the differences in the SAT and VAT but not in the MA between the SO and NOb groups. In a previous study [53], quantified body composition areas from MRI scans were strongly correlated to the same areas in CT images; thus, quantitative values of body composition contents among patients who underwent either one of the imaging examinations may be compared. Nevertheless, compared with CT images, MRI scans with multipurpose imaging methods such as T1-weighted images [16], T2-weighted images [53], proton MR spectroscopy (^1^H-MRS) [54], and spectral presaturation inversion recovery (SPIR) fat suppression [55,56] provide better contrasts in images of soft tissues including liver, kidney, muscle, and adipose tissues. In the present study, hepatic fibrosis in only SO patients positively correlated with traditional BMI and serum AST level, as well as the VAT measured from T1 THRIVE MR images. Therefore, the finding might be considered an indicator for evaluating the association between SO and hepatic fibrosis severity. For actual fat quantification, a study reported that ^1^H-MRS and MRI with the Dixon technique could provide reliable fat quantification using proton density water fraction and proton density fat fraction [57]. Further studies are necessary to validate the reliability of muscle/fat composition in large cohort populations using other specific imaging techniques.

This study has several shortcomings. First, this study focused on the association of hepatic fibrosis, and the two groups (NOb, SO) included middle-aged-matched sarcopenic subjects. Nevertheless, several studies have reported that aging including individual ability differences affects skeletal muscle strength, SMM, and physical performance [26,27]. However, it is a pity that the physical performance of patients was lacking, and also the skeletal muscle strength was lacking, in order to assess the level of sarcopenia in this study. Therefore, further study, including of the data, is needed to clarify the association. Moreover, the volumetric measurement of muscle and fat composition must be clarified as these factors can exhibit individual variations. These are dependent on multiple confounding factors, including sarcopenic severity, drinking, smoking, physical activity, and nutritional status [58,59]. However, in the present study, there was no consideration for drinking, smoking, physical activity, and nutritional status. Future studies are needed to clarify the pathological responses involved in sarcopenia and how these confounding factors affect SO. Second, BMI assessment using a Korean standard is the simplest method for evaluating obesity levels. However, it is restricted in assessing actual body composition contents because the values (not derived from the actual MM and FM) are indirect indices on the basis of body weight and height. In the criteria for overweight and obesity, the BMI cut-off values are different depending on geographical distributions as follows: in the Asia-Pacific region (>25.0 kg/m^2^), the western pacific regional office (WPRO) of WHO (>23.0 kg/m^2^), the World Health Organization (WHO) expert committee (25.0–29.9 kg/m^2^), and other countries [60]. Therefore, a standardized index will be more helpful in accurately measuring the actual amounts (muscle, fat) in patients with sarcopenia and/or SO. To overcome this shortcoming, an index derived from an image-based quantification program, such as sarcopenia software, can be a solution and can provide accurate body composition information (muscle and fat) for physicians. Third, instead of quantitative analysis in whole-body MRI, selecting a single L3 slice can provide a simple method for clinically quantifying body composition contents. This method may hold true in a cross-sectional study design. However in the longitudinal study design, it is limited because body weight change estimates from an L3 slice cannot replace whole-body assessments [17,49]. Therefore, quantitative analysis from a single-slice L3 MRI should be used cautiously for assessing body weight changes in patients. In addition, the estimation of SMM using MRI is based on mass rather than on tissue composition. The issue of quantifying the fat infiltration in muscle should be solved based on actual MM measurement in conjunction with hepatic deterioration by fat infiltration. A solution can be to introduce a multivoxel MRS technique for measuring fat infiltration [61].

## 5. Conclusions

This study demonstrated that patients with SO have larger SAT and VAT than patients with NOb. The hepatic fibrosis in patients with SO positively correlated with body visceral fat composition in combination with BMI and AST level. VAT quantified using sarcopenia software can be considered as an imaging biomarker of the hepatic fibrosis stage for SO. These findings will be useful for understanding the association between the hepatic manifestation of fibrosis and body fat composition in patients with SO.

## Figures and Tables

**Table 1:** Clinical characteristics in both NOb and SO groups according to hepatic fibrosis stage (F, METAVIR score).

	NOb (n = 28)				SO (n = 32)				*p*-Value * (NOb vs. SO)			
	F0 and F1 (n = 6)	F2 (n = 7)	F3 (n = 7)	F4 (n = 8)	F0 and F1 (n = 14)	F2 (n = 5)	F3 (n = 5)	F4 (n = 8)	F0 and F1 †	F2 †	F3 †	F4 †
Age (years)	58.0 ± 14.5				54.0 ± 14.9				0.290			
	53.8 ± 20.7	53.6 ± 12.4	59.4 ± 13.0	63.9 ± 12.4	50.9 ± 18.3	44.8 ± 12.2	63.0 ± 8.5	59.4 ± 8.2	0.718	0.268	1.000	0.505
BMI (Kg/m^2^)	22.3 ± 2.2				29.1 ± 3.4				<0.001			
	23.3 ± 2.1	20.5 ± 2.3	22.7 ± 2.0	22.8 ± 1.9	28.0 ± 2.6	28.9 ± 2.9	28.6 ± 2.2	31.6 ± 4.8	<0.001	0.003	0.003	<0.001
Albumin (g/dL)	3.95 ± 0.12				4.02 ± 0.11				0.705			
	3.84 ± 0.23	3.95 ± 0.25	4.04 ± 0.15	3.98 ± 0.27	4.05 ± 0.48	4.30 ± 0.25	4.80 ± 0.21	3.84 ± 0.13	0.429	1.000	0.333	0.234
ALT (IU/L)	88.9 ± 32.0				75.0 ± 19.4				0.713			
	47.6 ± 20.0.	99.2 ± 49.9	276.4 ± 205.9	41.8 ± 11.9	94.7 ± 34.1	126.4 ± 64.2	44.5 ± 29.5	28.4 ± 7.4	0.438	0.662	0.857	0.328
AST (IU/L)	138.0 ± 47.6				68.7 ± 7.9				0.293			
	49.8 ± 10.2	163.0 ± 68.4	276.4 ± 225.5	135.6 ± 58.6	49.4 ± 7.3	83.2 ± 32.1	59.0 ± 22.6	95.3 ± 16.4	0.743	0.429	0.857	0.573
ALP (IU/L)	174.0 ± 24.2				195.5 ± 29.2				0.572			
	124.0 ± 31.2	188.8 ± 65.5	276.8 ± 56.1	129.8 ± 22.8	179.3 ± 28.5	150.0 ± 55.5	82.0 ± 24.0	281.1 ± 72.3	0.190	0.931	0.190	0.054
CRP (mg/dL)	3.68 ± 0.92				2.02 ± 0.61				0.198			
	7.65 ± 3.31	3.04 ± 0.99	1.49 ± 0.48	4.05 ± 2.17	1.09 ± 0.32	1.55 ± 0.34	4.58 ± 0.45	2.22 ± 0.92	0.333	0.400	0.500	0.629
Fasting glucose (IU/L)	143.4 ± 21.8				114.4 ± 13.2				0.255			
	144.7 ± 29.9	115.8 ± 18.0	135.7 ± 13.4	165.2 ± 57.0	125.4 ± 19.0	112.8 ± 34.7	192.0 ± 20.3	94.3 ± 2.1	0.497	0.905	0.593	0.114
GGT (U/L)	122.1 ± 19.8				89.9 ± 24.4				0.307			
	152.6 ± 54.7	170.8 ± 42.8	102.6 ± 37.8	84.8 ± 28.2	48.3 ± 12.8	112.0 ± 41.7	125.0 ± 15.9	147.4 ± 78.4	0.127	0.421	0.333	0.622

Biochemistry data are presented as mean ± SEM. Abbreviations—BMI: body mass index; ALT: alanine aminotransferase; AST: aspartate aminotransferase; ALP: alkaline phosphatase; CRP: C-reactive protein; GGT: gamma-glutamyl transferase. * The difference between NOb and SO groups was analyzed using the independent two-sample *t*-test. † The difference between NOb and SO within each fibrosis stage was analyzed using the Mann–Whitney U test.

**Table 2:** Comparisons of body composition contents between NOb and SO groups.

	NOb (n = 28)				SO (n = 32)				*p*-Value * (NOb vs. SO)			
	F0 and F1 (n = 6)	F2 (n = 7)	F3 (n = 7)	F4 (n = 8)	F0 and F1 (n = 14)	F2 (n = 5)	F3 (n = 5)	F4 (n = 8)	F0 and F1 †	F2 †	F3 †	F4 †
MA (mm^3^)	12,376 ± 2364				13,795 ± 3422				0.064			
	13,122 ± 3059	11,924 ± 2405	13,250 ± 2231	11,448 ± 1793	15,687 ± 3394	15,314 ± 4196	12,730 ± 3831	14,200 ± 2603	0.353	0.639	0.432	0.234
SAT (mm^3^)	14,885 ± 4361				23,119 ± 8563				<0.001			
	16,468 ± 3580	14,986 ± 4979	14,936 ± 5761	13,567 ± 3217	24,186 ± 8903	23,078 ± 7270	17,909 ± 4720	24,532 ± 10,520	0.091	0.106	0.432	0.021
VAT (mm^3^)	8126 ± 4401				15,970 ± 5528				<0.001			
	9181 ± 6974	7323 ± 3104	7172 ± 4378	8872 ± 3485	12,413 ± 4687	14,950 ± 2759	22,180 ± 2475	18,951 ± 4790	0.207	0.005	0.003	0.001
MA/SAT ratio	0.94 ± 0.43				0.68 ± 0.30				0.009			
	0.86 ± 0.37	0.88 ± 0.31	1.09 ± 0.67	0.92 ± 0.36	0.73 ± 0.32	0.71 ± 0.25	0.79 ± 0.41	0.68 ± 0.35	0.274	0.268	0.432	0.083
MA/VAT ratio	2.00 ± 1.18				1.06 ± 0.76				<0.001			
	2.22 ± 1.70	1.95 ± 0.92	2.32 ± 1.08	1.60 ± 1.13	1.46 ± 0.99	0.98 ± 0.29	0.53 ± 0.18	0.73 ± 0.21	0.397	0.018	0.003	0.001
MA/(SAT+VAT)	0.59 ± 0.25				0.38 ± 0.17				<0.001			
	0.52 ± 0.15	0.58 ± 0.20	0.68 ± 0.37	0.56 ± 0.24	0.45 ± 0.21	0.39 ± 0.11	0.31 ± 0.13	0.32 ± 0.09	0.274	0.048	0.010	0.007

Data are presented as mean ± SD. Abbreviations—MA: muscle area; NOb: non-obesity; SAT: Subcutaneous adipose tissue; SO: sarcopenic obesity; VAT: visceral adipose tissue. * The difference between NOb and SO groups was analyzed using the independent two-sample *t*-test. † The difference between NOb and SO within each fibrosis stage was analyzed by using Mann–Whitney U test.

**Table 3:** Comparisons of body composition contents between NOb and SO groups.

	Correlation Coefficients	All	NOb	SO	*p*-Value * (Two-Tailed)		
Factors		(n = 60)	(n = 28)	(n = 32)	All	NOb	SO
Demographical factors							
Age (years)		0.240	0.289	0.198	0.065	0.136	0.276
BMI (body mass index, kg/m^2^)		−0.017	0.054	0.443	0.896	0.783	* 0.011
Blood chemistry factors							
Albumin (g/dL)		0.002	0.226	−0.254	0.992	0.288	0.325
Alanine aminotransferase (ALT, IU/L)		−0.260	−0.143	−0.344	0.075	0.504	0.100
Aspartate aminotransferase (AST, IU/L)		0.324	0.319	0.415	* 0.025	0.129	* 0.044
Alkaline phosphatase (ALP, IU/L)		0.104	0.070	0.164	0.488	0.746	0.455
C-reactive protein (CRP, mg/dL)		0.041	−0.311	0.540	0.863	0.326	0.168
Fasting glucose (IU/L)		−0.062	−0.083	−0.159	0.734	0.761	0.543
gamma-glutamyl transferase (GGT, U/L)		0.054	−0.284	0.251	0.733	0.188	0.300
Body composition content factors							
Muscle area (MA, mm^3^)		−0.198	−0.156	−0.191	0.129	0.427	0.296
Subcutaneous adipose tissue (SAT, mm^3^)		−0.190	−0.263	−0.001	0.146	0.176	0.998
Visceral adipose tissue (VAT, mm^3^)		0.183	0.095	0.653	0.162	0.630	*** <0.001
MA/SAT ratio		0.038	0.086	−0.126	0.775	0.664	0.491
MA/VAT ratio		−0.244	−0.124	−0.605	0.060	0.529	*** <0.001
MA/(SAT+VAT) ratio		−0.125	−0.001	−0.416	0.341	0.997	* 0.018

* The correlation coefficients between METAVIR pathologic stage and each risk factor were analyzed using linear polynomial correlation. Statistical significance is indicated as follows: * *p* < 0.05, and *** *p* < 0.001.

## Data Availability

The datasets used and/or analyzed during the current study are available from the corresponding author on reasonable request.
